# Discovery of sensorineural hearing loss and ossicle deformity in a Chinese Li nationality family with spondyloepiphyseal dysplasia congenita caused by p.G504S mutation of *COL2A1*

**DOI:** 10.1186/s12920-021-01020-y

**Published:** 2021-06-28

**Authors:** Kan Wu, Zhumei Li, Yuhua Zhu, Xiaocheng Wang, Guohui Chen, Zhaohui Hou, Qiujing Zhang

**Affiliations:** 1 Department of Otolaryngology-Head and Neck Surgery, Chinese PLA Institute of Otolaryngology, Chinese PLA General Hospital, Medical School of Chinese PLA, 28 Fuxing Road, Beijing, 100853 China; 2 Centre of Clinical Aerospace Medicine, School of Aerospace Medicine, Key Laboratory of Aerospace Medicine of Ministry of Education, Air Force Medical University, Xi’an, 710032 Shanxi Province China; 3 Department of Otolaryngology-Head and Neck Surgery, Wanning People’s Hospital, Wanning, 571500 Hainan Province China

**Keywords:** Spondyloepiphyseal dysplasia congenita, *COL2A1*, Sensorineural hearing loss, Variation, Genetics

## Abstract

**Background:**

Spondyloepiphyseal dysplasia congenita (SEDC) is an autosomal dominant chondrodysplasia characterized by disproportionate short stature, abnormal epiphyses, and flattened vertebral bodies. *COL2A1* has been confirmed as the pathogenic gene. Hearing loss represents an infrequent manifestation for 25–30% of patients with SEDC. The characteristics of the hearing impairment were rarely documented.

**Methods:**

Audiological, ophthalmic, imaging examinations were conducted on the family members. The whole exome sequencing (WES) was performed to detect the candidate gene, and the Sanger sequencing was used to confirm the causative variation.

**Results:**

*COL2A1* c.1510G>A (p.G504S), a hot spot variation, was identified as the disease-causing mutation of the Chinese Li nationality family with SEDC. This variation was co-segregated with the SEDC phenotype in the family and was absent in the 1000 Genomes Project, ESP and ExAC. Clinically, several manifestations were first demonstrated in SEDC patients caused by p.G504S, including sensorineural hearing loss, auditory ossicles deformity, retinal detachment, sacrum cracked and elbow and wrist joints deformity. Other classical SEDC manifestations such as bones and joints pain, midfacial dysplasia, disproportionate short stature, spinal deformity, thoracocyllosis, coxa arthropathy, myopia and waddling gait were also showed in the family patients.

**Conclusion:**

We first identified the mutation p.G504S in *COL2A1* gene as the pathogenesis in a Chinese Li nationality family and reported the correlation between p.G504S and atypical clinical phenotypes including sensorineural hearing loss, auditory ossicles deformity, retinal detachment, sacrum cracked and elbow and wrist joints deformity. Our findings would extend the phenotypic spectrum of SEDC and deepen clinicians' understanding of genotype–phenotype correlation of the disease.

**Supplementary Information:**

The online version contains supplementary material available at 10.1186/s12920-021-01020-y.

## Background

Spondyloepiphyseal dysplasia congenita (SEDC, MIM#183900) is an autosomal dominant chondrodysplasia characterized by disproportionate short stature, abnormal epiphyses, and flattened vertebral bodies. With the low incidence of approximately 3.4 per million people, SEDC is a rare clinical subtype of type II collagenopathies. The subtype was firstly reported by Spranger and Wiedemann in 1966.

*COL2A1* (OMIM: 120140) has been identified as the pathogenic gene of SEDC, which is located at 12q13.11 with the length of 31,510 bp. It encodes alpha-1(II) chain of type II procollagen, which is composed of 1487 amino acids. Three α-1 chains folded together to form a triple-helix configuration of the procollagen homotrimer. Mature collagen molecules processed through shear, modification and other necessary steps in the extracellular matrix form a covalently cross-linked fibrillar network, providing tensile strength for the connective tissues.

To date, Type II collagenopathies can be classified into at least 16 definite disorders [1]. Among these disorders, SEDC is mainly manifesting spinal deformity. The skeletal system abnormalities show up at birth and worse with age. Other manifestations of SEDC include myopia and/or retinal degeneration detached, cleft palate. Hearing loss represents an infrequent manifestation for 25–30% of patients with SEDC. Most papers reported a loss on the order of 30–70 dB at the higher frequencies (4 to 8 kHz) [2].

In the present study, a Chinese Li nationality family with SEDC was firstly reported around the world and the *COL2A1* p.G504S was identified as the disease-causing mutation. To date, there have been 632 mutations of the *COL2A1* reported in the DVD that are pathogenetic and responsible for SED and other subtypes of skeletal dysplasia. (DVD: http://deafnessvariationdatabase.org/gene/COL2A1, 2021.03.12). The mutation p.G504S, as a hot spot, was firstly reported to be associated with sensorineural hearing loss and ossicle deformity in a SEDC family in this study. This finding would extend the phenotypic spectrum of SEDC and deepen clinicians' understanding of genotype–phenotype correlation of the disease. It also laid a foundation of the further study of genetic counselling, fertility guidance and gene function.

## Methods

### Subjects

The Family 1,908,322, living in Lingshui Li Autonomous County, Hainan province, was a local family that had been passed down for 3 generations. The family members were all of Li nationality. The study was approved by the Committee of Medical Ethics of Chinese People’s Liberation Army (PLA) General Hospital. Written consents were obtained from all the participants aged over 18 and from the next of kin on behalf of the minors/children’s participants. All the experiments in this study were performed in accordance with the Declaration of Helsinki and other relevant guidelines and regulations.

The proband, a 21-year-old deaf-mute female (II3), was admitted to the Department of Otolaryngology-Head and Neck Surgery of Hainan Branch of Chinese PLA General Hospital in July 2019. She underwent detailed inquiry, physical examination and relevant auxiliary examinations in visual, auditory and skeletal systems. 10–20 ml peripheral venous blood samples were taken for DNA extraction and test. Other family members (father, mother, older sister, younger sister and nephew of the proband) (I1, I2, II2, II4, III1) also took part in the experiment and underwent relevant examinations. Their peripheral blood samples were also taken.

### Medical history collection and physical examination

Detailed medical history was collected using the standard questionnaire, which included general information, medical history of present illness, past history, personal history, birth history, family history, among others. Physical examination was conducted by a team of experienced specialists and focused on the musculoskeletal, auditory, and visual systems.

### Auxiliary examination

Diagnostic audiometer (Madsen Conera, Denmark), headphone with air guide (TDD-39, Denmark) and bone guide (b-71, Denmark), and acoustic conductivity detector (Madsen OTO Flex100, Denmark) were used for pure-tone audiometry (PTA) and acoustic immitance. Instruments were regularly calibrated in accordance with international standards. Audiological tests were conducted in standard soundproof rooms. The average hearing threshold was calculated as the pure tone air-conduction averages of the air-conduction thresholds at 0.5, 1, 2, and 4 kHz. Hearing loss levels were graded into the following categories basis the pure tone air-conduction averages: subtle (16–25 dB), mild (26–40 dB), moderate (41–55 dB HL), moderately severe (56–70 dB HL), severe (71–90 dB HL), and profound (> 91 dB HL).

All the family patients (I2, II2, II3, III1) received spine and hip joint X-ray. The adult patients (I2, II2, II3) also received temporal bone CT scans, PTA, acoustic immitance and ophthalmic tests including optometry, intraocular pressure, anterior segment and fundus colour photography. All the abnormalities evidences of subjects were identified by a team of experienced specialists and technicians.

### Genetic test

The whole-exome sequencing (WES) was conducted on DNA samples of the patient II3 and I2 with Illumina HiSeq2500 platform. The target area coverage was 99.73% (II3) or 99.52% (I2). The average sequencing depth for target region was 138.90X (II3) or 180.52X (I2), with 98.72% (II3) or 98.89%(I2) of the target sequence reaching up to 20X. After base identification, raw sequencing reads were mapped against the human reference genome (NCBI37/hg19). Variants were filtered against databases including ClinVar, OMIM and HGMD. Several large-scale population-based databases were also used to eliminate high-frequency variations in the normal population, including EPS6500, 1000 Genomes Project, GnomAD, ExAC, dbscSNV, MatEntScan, dbNSFP. Further bioinformatics analyses were carried out using the SIFT, PolyPhen-2, Mutationtaster, PhyloP and GERP online tools to predict the function of potential pathological variation. Sanger sequencing was performed on DNA samples of other family members to determine whether the potential variation in the candidate gene co-segregated with the phenotype in the family or not. A pair of PCR primers designed to validate the potential causative variation in exon 23 of *COL2A1* were as follows: 5′-GAGGATGACATGCGGAAAAGTC-3′, 5′-CAGTTGGATCTTTAGCCCCTCT-3′. All the family members were checked for mitochondrial genome variations associated to hearing loss, including m.1095T>C, m.1494C>T, m.1555A>G, associated with deafness.

## Results

### Clinical evaluation

The proband (II3) was a 21-year-old deaf-dumb female. At the age of 2 to 3 years, she began to suffer from growth retardation and disproportionate short neck and trunk. At the time of physical examination, she was 125 cm tall and weighed 30 kg. She had bones and joints pain, midfacial dysplasia, barrel chest, kyphoscoliosis, elbow and wrist joints deformity and unsteady waddling gait. History of limb trauma was denied. PTA showed profound hearing impairment in bilateral ears. The spine X-ray indicated that the thoracic spine was curved to the left and the sacrum was cracked. Hip joint X-ray showed significant abnormalities in bilateral hip joints, including aseptic inflammation, subluxation, acetabular fossa shallowing, femoral heads collapsing, articular surface destruction, low-density shadows under the articular surface, unclear boundary, narrowing of joint space, and femoral neck shortening. Temporal bone CT scan revealed that the structure of bilateral auditory ossicles was abnormal with disproportionate short incus bodies. The optometry showed myopia in both eyes. The fundus colour photograph demonstrated abnormalities in fundus tissue, including arc-shaped atrophy spots around the optic papilla, strong white reflection of the nerve fibre layer, stiff macular vascular arch, and yellow-white exudative changes. Intraocular pressure and anterior segment were normal.

The mother of the proband (I2), a 51-years-old female, had short stature since childhood. At the time of physical examination, she was 133 cm tall and weighed 35 kg. She had bones and joints pain, disproportionate short neck and trunk, midfacial dysplasia, kyphosis with limited spinal movement and unsteady waddling gait. PTA showed her bilateral mild high-frequencies hearing loss. The spine X-ray revealed kyphosis. Hip joint X-ray showed aseptic inflammation of bilateral hip joints, collapsing of bilateral femoral heads, unsmooth femoral head articular surfaces with increasing density, quasi-circular and low-density shadows under the articular surface, unclear boundary, narrowing of joint space, and femoral neck shortening. Temporal bone CT scan and all the ophthalmic tests were normal.

The older sister (II2) of the proband was 28-years-old. She had a short stature with disproportionate short neck and trunk since she was a child. She was 143 cm tall and weighed 42 kg when she was taken examination. Midfacial dysplasia, bones and joints pain and unsteady waddling gait were also present. Hip joint X-ray revealed ischemia of femoral heads, hyperplasia and sharpened edges of femoral heads, unsmooth acetabular and femoral head articular surfaces with nonhomogeneous density, patchy and slightly low-density shadows, and narrowing of joint space. The PTA, temporal bone CT scan and all the ophthalmic tests were normal.

The nephew of the proband (III1) was a 3-years-old boy with normal hearing and speech development, while having growth retardation. He was 75 cm tall and weighed 8.5 kg when he was taken physical examination. Midfacial dysplasia, disproportionate short neck and trunk, and pigeon chest could be detected by visual examination. He had no noticeable bones or joints pain, and had no difficulty in walking. Due to his inability to cooperate, he did not undergo auxiliary examination including PTA, temporal bone CT scan and ophthalmic examinations.

The 20-years-old younger sister (II4) of the proband was 162 cm tall and weighed 55 kg. No abnormalities were found in the auditory, visual and skeletal systems.

**Fig. 1:**
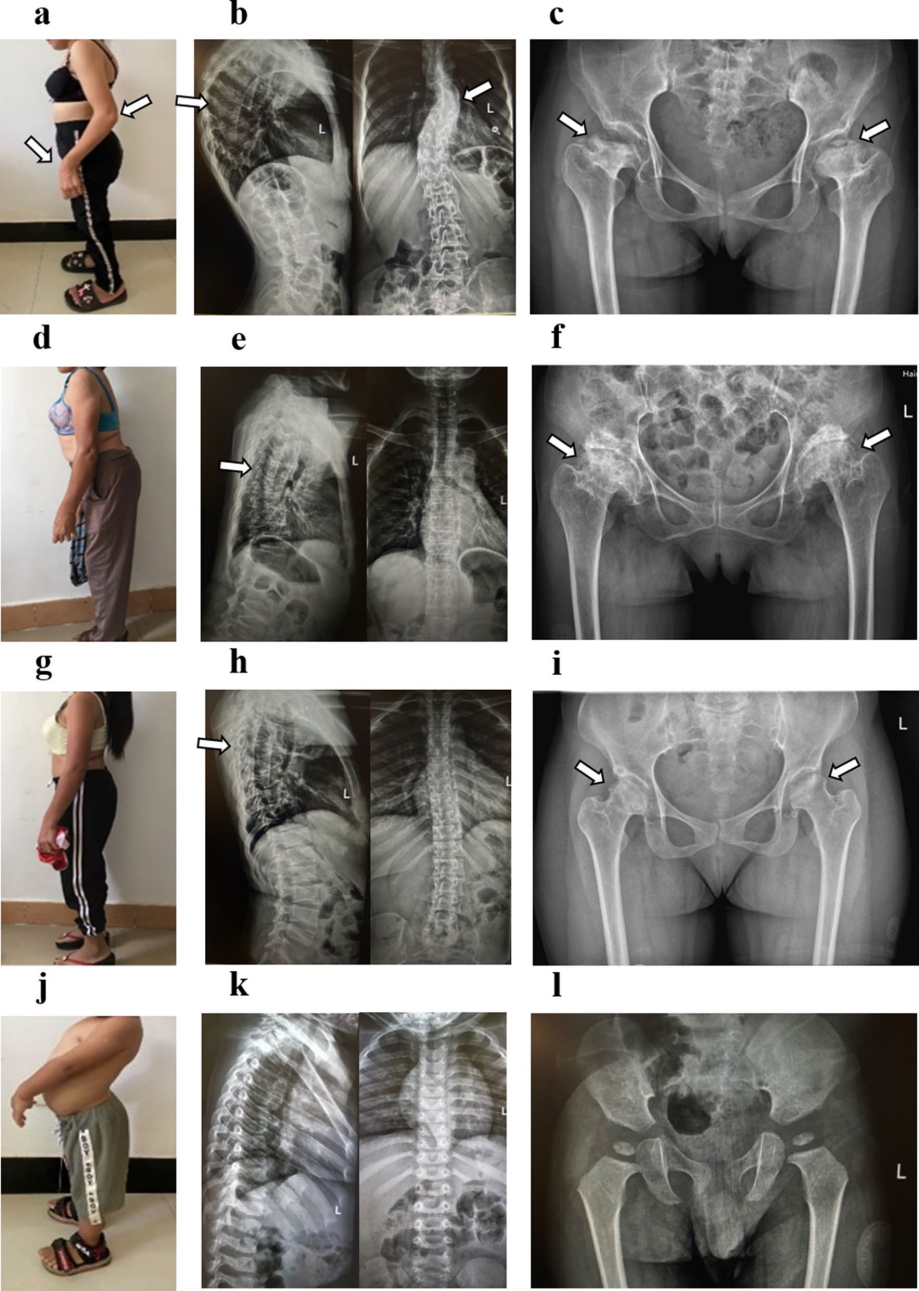
General appearance photographs and skeletal system radiographs of patients (II3: **a**–**c**, I2: **d**–**f**, II2: **g**–**i**, III1: **j**–**l**) in Family 1,908,322. All the patients presented with disproportionate short neck and trunk and had no deformities in fingers (**a**, **d**, **g**, **j**). The proband (II3) presented with barrel chest, deformity of elbow and wrist joints (**a**). The youngest patient (III1) presented with pigeon chest and had normal fingers (**j**). The spine X-ray photographs of adult patients revealed kyphoscoliosis (**b**) or kyphosis (**e**, **h**). The hip joint X-ray showed bilateral femoral heads ischemia or aseptic inflammation, bilateral hip joints subluxation, bilateral acetabular fossa shallowing, bilateral femoral heads collapsing, bone destruction on articular surface, irregular and low-density shadows under the articular surface, unclear boundary, narrowing of joint space, and shortening of femoral neck (**c**, **f**, **l**). The radiographs of III1 were normal (**k**–**l**)

**Fig. 2:**
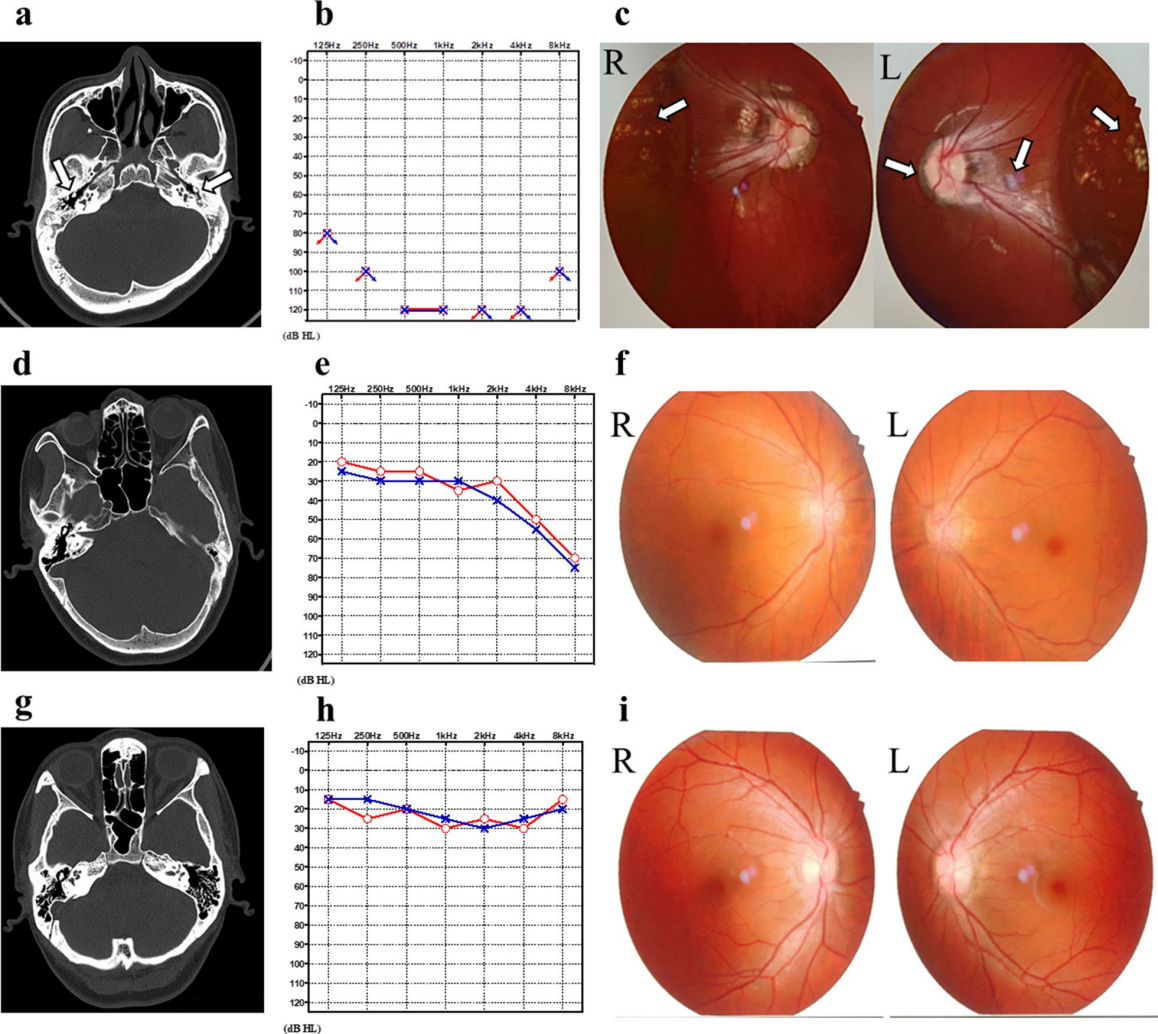
The temporal bone CT scans, pure-tone audiometry curves and fundus colour photographs of adult patients (II3: **a**–**c**, I2: **d**–**f**, II3: **g**–**i**) in Family 1,908,322. The temporal bone CT of the proband (II3) revealed that the structure of bilateral auditory ossicles was abnormal with disproportionate short incus bodies (**a**). Pure tone audiometry curves showed that the proband (II3) suffered from profound hearing impairment in all frequencies (**b**) and her mother (I2) had mild hearing loss in high frequencies (**e**). The fundus colour photograph of the proband (II3) demonstrated that arc-shaped atrophy spots can be seen around the optic papilla, strong white reflection of the nerve fibre layer can be seen, the macular vascular arch is stiff, and a lot of yellow-white exudative changes (**c**). The temporal bone CT scans and fundus colour photographs of I2 and II3 were normal (**d**, **f**, **g**, **i**). The pure tone audiometry of II3 was normal (**h**)

All the family members were Li nationality and denied the history of ototoxic drug abuse and noise exposure. There was no consanguineous marriage in the family. All the family members had normal intelligence and normal birth history. Their birth weight and length had not been recorded. The adult patients in this family were taller than the average height for SEDC adult patients, which was regarded as 115.50 cm [3]. The pain of bones and joints was progressively aggravated among the adult patients. All the family members’ bilateral tympanic membranes were clear by electro-otoscope examination. Other SEDC manifestations such as cleft palate, short-toe deformity and flattened vertebral bodies were not presented in the family patients (see Figs. 1 and 2).

### Genetic tests

**Fig. 3:**
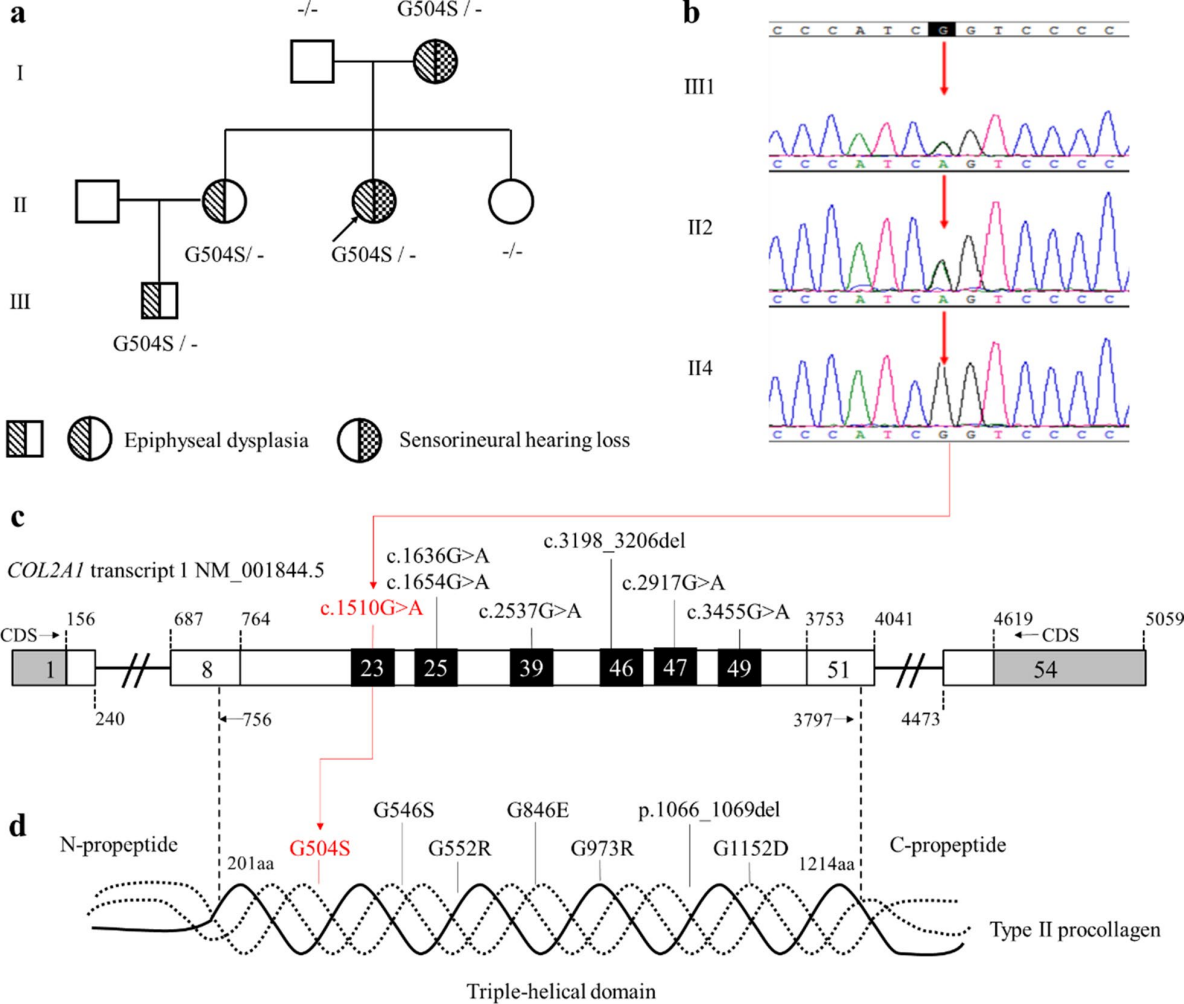
Pedigree, sequencing peak map and location of the variation in gene and COL2A1 protein. The proband was indicated by an arrow. G504S-minus signs represented heterozygous *COL2A1* variation carriers, double-minus signs, wild type. Slash filled symbols for males (squares) and females (circles) represented affected individuals with epiphyseal dysplasia, and spots, with sensorineural hearing loss. White filled symbols for squares and circles represented the normal family members (**a**). Sanger sequencing confirmed the co-segregation of the pathogenic *COL2A1* variation with the phenotype in Family 1,908,322 (**b**). Structure of *COL2A1* transcript 1 (NM_001844.5) with 54 exons. Pathogenic variation c.1510G>A was located in exon 23 and was marked with red and five previously reported spondyloepiphyseal dysplasia congenita (SEDC) accompanied causative variations associated with sensorineural hearing loss (SNHL) were distributed on several different exons (**c**). The triple-helical configuration of the type II procollagen was assembled from three α-1 chain encoded by *COL2A1* and all of reported variations causing SEDC accompanied with SNHL are distributed within the triple-helical domain rather than C or N terminal propeptide (**d**). Reference website for drawing the structure diagram of the gene and procollagen: https://www.ncbi.nlm.nih.gov/

Exons and adjacent intronic regions were captured and sequenced using WES for the patient II3 and I2. After the databases filtration and bioinformatics analyses on the data, a heterozygous variation c.1510G>A (p.Gly504Ser) in exon 23 of the *COL2A1* gene (NM_001844.4) was detected. It was predicted to be the potential disease-causing variant of the Chinese Li nationality family. We didn’t find any known pathogenic deafness-related variants in mitochondrial genome of the family members. Sanger sequencing confirmed the co-segregation of the candidate *COL2A1* variation with the phenotype in Family 1,908,322 (see Fig. 3).

### Variation detection and analysis

The average sequencing depth and coverage for targeted region were satisfied with the requirements for calling SNPs and InDels (Additional File 1: Table S1). In the filtering process of variants of II3, firstly, using genotype frequency, including 1000 human genome dataset, ExAC, ESP6500, GnomAD and BGI in-house Database, less than 0.01 as the filtering criteria, 336 heterozygous mutations and 9 homozygous mutations were filtered out from the annotated variants (Additional File 1: Table S1–S3). Secondly, the annotations in databases were used to score the location and impact of mutations. There were 166 heterozygous and 2 homozygous functional variations including tran ablation/stop gained/stop lost/frameshift /missense/inframe insertion/deletion were filtered out. Thirdly, 52 heterozygous variants were predicted to be deleterious by software such as SIFT, MutationTaster, Polyphen2, Phylop, GERP et al. Among these variants, *COL2A1* G504S was the only one which was associated with SEDC and hearing loss by checking for disease and gene-specific databases (see Additional File 2: Fig. S1 and Additional File 1: Table S2). The filtering analysis process of variants of I2 was similar to that of II3 (see Additional File 3: Fig. S2 and Additional File 1: Table S2). The variation had been reported to be the pathogenic variation of SEDC, regarded as a hot spot variation [4–9]. The genotype frequency in the 1000 Genomes Project, ESP and ExAC was less than 0.001. The population frequency in databases such as ESP6500, Thousands, EXAC, GnomAD was 0. The variant occurred within the highly evolutionarily conserved region across different species and was predicted to be deleterious with Condel, PolyPhen 2, Mutationtaster and SIFT programs (Additional file 1: Table 3). We ruled out other possible heterozygous, homozygous nor compound heterozygous variations by WES (Additional File 1: Table S4–S5). And all the family members didn’t carry any known deafness-related mitochondrial variants. We had found sufficient evidence (PS1 + PM1 + PM2 + PP2 + PP3 + PP1-Moderate + PP4 + PS4-Support) according to the American College of Medical Genetics and Genomics Guideline [10] to prove *COL2A1* c.1510G>A to be the pathogenic variant of Family 1,908,322.

## Discussion

Hearing loss represents an infrequent manifestation of patients with SEDC. Only six mutations of *COL2A1* gene had been reported to simultaneously cause hearing loss and scoliosis [4, 11–15] (Table 1). Hearing loss was absent in patients’ phenotypic spectrum caused by p.G504S of *COL2A1* before this study (Table 2). In this family, the proband (II3) suffered from profound sensorineural hearing loss. Her mother (I2) had bilateral mild sensorineural hearing loss. There were considerable differences in the degree of their hearing loss. We did not find any possible deafness-causing variations except G504S in *COL2A1* using WES and checking for mitochondrial genome. Other known genetic causes were ruled out and the patient had a normal birth and denied history of ototoxic drug abuse and noise exposure. In previous studies [4, 11–15], phenotypic diversity was prevalent in *COL2A1*-related diseases, especially SEDC combined with hearing impairment. Hearing loss varied from mild to profound (Table 1). Actually, the proband had more severe deformities and dysfunction in visual, auditory and skeletal systems than other patients within the family. To be more specific, myopia, ocular fundus changes, auditory ossicles dysplasia and deformity of elbow and wrist joints were only occurred in the proband. Moreover, the height of the proband was shorter. And her scoliosis and hip joints abnormality such as femoral heads disease were more pronounced than the corresponding phenotypes of patient I2 and II2. Therefore, the difference of hearing loss degree was more likely one of phenotypic spectrum diversity associated to the common genetic pathogenesis, which was identified to be the variant G504 in *COL2A1*. Possible reasons for phenotypic differences within the family include: (1) incomplete penetrance in this family (2) randomness in developmental processes such as gene expression.

**Table 1:** Summary of the characteristics of SEDC* patients with hearing loss caused by *COL2A1* variations

References	Gender	Age	Origin	Family members	Deafness/SEDC	Nucleotide	Amino acid	Inherited pattern	Exon	Descriptions of hearing loss in the literature**
Zheng et al., (2020)	M	33	Chinese	3	1/2	c.1654G>A	G552R	AD	25	Mild
Sobetzko et al., (2000)	M	7	European	12	1/1	c.2917G>A	G973R	de novo	47	Moderate sensorineural Hearing deficit causing speech delay
Xu et al., (2014)	M	42	Chinese	8	1/5	c.1636G>A	G546S	AD	25	Conductive hearing loss
Zhang et al. (2011)	F	6	Chinese	22	1/2	c. 3455 G>A	G1152D	AD	49	Decreased hearing in both ears
Nishimura et al., 2005	M	6y8m	Japanese	Sporadic	Sporadic	c.2537G>A	G846E	Sporadic	39	Mild
Ideura et al. (2019)	NA	NA	Japanese	3	2/2	c.3198_3206del	p.1066_1069del	AD	46	Severe to profound

^*^SEDC: Spondyloepiphyseal dysplasia congenita, MIM#183,900. **All the six studies descripted hearing impairment according to the patients’ chief complaint rather than undergoing audiological examinations. ***NA: not available

**Table 2:** Clinical characteristics of spondyloepiphyseal dysplasia congenita patients carried variation p.G504S in *COL2A1*

Family number	II3	I2	II2	III1	Family 1	Family 2	Family 3	Family 4	Family 5	Family 6	Family 7
Country (Ethnicity)	China (Li)	China (Li)	China (Li)	China (Li)	China (Han)	China (Han)	China (Han)	Japan	Japan	Japan	China (Han)
patients/family size	4/7				1/3	9/21	9/35	1/1	1/1	1/4	1/1
Generations	3 (Het)				De novo	4 (Het)	4 (Het)	Sporadic	Sporadic	De novo	sporadic
Gender	F	F	F	M	M	F	F	M	M	F	M
Age	21y	51y	28y	3y	13y	27 m	53y	24y	60y	2y	9y
Height (cm)	125	133	143	75	116.5	80.5	NA	137.2	151	72.6	NA
Weight (kg)	30	35	42	8.5	NA	NA	NA	NA	NA	10.6	NA
Intelligence	−	−	−	−	−	NA	NA	NA	NA	NA	NA
Bone or joint pain	+	+	+	−	+	−	NA	NA	NA	NA	NA
Waddling gait	+	+	+	−	+	−	+	NA	NA	NA	NA
Short trunk	+	+	+	+	+	+	+	+	+	+	+
Short neck	+	+	+	+	+	NA	+	NA	NA	NA	NA
Midfacial dysplasia	+	+	+	+	−	−	NA	NA	NA	−	NA
Cleft palate	−	−	−	−	−	−	−	−	−	−	NA
Thoracocyllosis	+	−	−	+	−	−	−	−	−	−	NA
Scoliosis	+	−	−	−	+	−	+	+	+	−	NA
Kyphosis	+	+	+	−	−	−	−	+	+	−	NA
Platyspondyly	−	−	−	−	+	−	+	NA	NA	+	NA
Elbow deformity	+	−	−	−	−	NA	NA	NA	NA	NA	NA
Wrist deformity	+	−	−	−	−	NA	NA	NA	NA	NA	NA
Acetabulum abnormality	+	+	+	−	+	−	+	NA	NA	+	NA
Femoral head disease	+	+	+	−	+	−	+	NA	NA	+	NA
Sacrum cracked	+	−	−	−	−	−	−	NA	NA	−	NA
Myopia	+	−	−	−	−	−	−	−	−	+	NA
Retinal detachment	+	−	−	−	−	−	−	−	−	NA	NA
Hearing impairment	+	+	−	−	−	−	−	−	−	−	NA
ossicles deformity	+	−	−	−	NA	NA	NA	NA	NA	NA	NA
Variations	G504S	G504S	G504S	G504S	G504S	G504S	G504S	G504S	G504S	G504S/G612A	G504S
Literature	This study	This study	This study	This study	Xu et al. 2020	LH et al. 2012	Xia et al. 2007	Nishimura et al. 2005	Nishimura et al. 2005	Kawano et al. 2015	Zhang et al. 2015

^*^NA: not available, +: present, −: absent

The application and records of audiological tests were generally scarce in previous literatures [4, 11–15]. Hearing impairment descriptions were mostly based on the patients’ chief complaints rather than audiological examinations. The degrees of hearing loss varied from mild to profound and most patients were suffered from sensorineural hearing loss. In addition, Dahiya et al. [2] describe a case of a patient with mixed hearing loss that was likely attributable to stapes fixation. It was also believed that type II collagen was present in the cartilage that gave rise to the ossicles, which could account for the malformation of ossicles of the proband in the present study. Compared with SEDC, another subtype of *COL2A1*-related diseases type I of Stickler syndrome (STL1) had a much higher incidence of SNHL, from 50 to 75%. These STL1 patients suffered from mild-to-moderate high-frequencies SNHL since their childhood and their hearing loss progressed more slowly [16–18].

The mechanism for the auditory system abnormality associated with *COL2A1* had not been fully uncovered. In humans, *COL2A1* gene was expressed in cartilage cell, vitreous humor, intervertebral disc and inner ear structures. In the gerbil model, type II collagen in the connective tissue and tectorial membrane of the organ of Corti combined with radiating fibres to form a highly structured matrix together with type V and type IX collagen, which helped to enhance the hardness of tectorial membrane and enabled it to withstand the physical stress caused by sound conduction [19].

The mutation p.G504S of *COL2A1* had been reported as the pathogenesis of 7 unrelated families before this study (see Table 2). The ethnicity of the patients was either Han Chinese or Japanese. The patients in this study were the Li ethnic group from Hainan Province, southern China. Besides hearing impairment, atypical manifestations in the proband, including auditory ossicles deformity, retinal detachment, midfacial dysplasia, thoracocyllosis, sacrum cracked and elbow and wrist joints deformity, had never been demonstrated in SEDC patients caused by mutation p.G504S. On the other hand, several manifestations, including disproportionate short stature and neck, spine malformation including scoliosis and kyphosis, acetabulum abnormal, and femoral head disease, were common symptoms among different families. The proband from Family 1 [9] had a pain, limitation in hip mobility and abnormal gait from 5 years old and the symptoms increased with age. Short heights, lower extremities and spines abnormal were his main clinical manifestations. All affected individuals presented with similar clinical features in Family 3 [5] rather than significant diversity in the Li nationality family in this study. In family 4 and family 5, the p.G504S was responsible for milder skeletal phenotypes, called subtypes SEDC-M (SEDC-mild) and SEDT (late-onset SED), than the phenotype in this study [4].

The *COL2A1*-related spectrum diseases demonstrate significant genetic heterogeneity. Despite numerous studies had been done, a clear and directional correlation between genotype and phenotype has not yet been established. Several speculations were made in the previous literatures. And a few specific genotype–phenotype relationships have been described. For example, splicing variations was considered to be more likely to cause severe hearing loss and patients with splicing variations had a higher proportion of hearing aid [20]. Mutations located in the C-terminal (carboxyl terminal) propeptide of the type II procollagen could also cause hearing impairment. Terhal et al. [21] reported that half of 6 patients with a C-terminal propeptide variation complained of hearing loss, and one required hearing aids. Moreover, variations in the C-terminal domains seemed more likely to produce severe spinal deformity, coxa arthropathy, cleft palate and hearing impairment, because the collagen triple helix would fold in a C-to-N terminal direction [22]. In the present study, the disease-causing variant was not in the C-terminal propeptide and the other five variations associated with SEDC and SNHL simultaneously were evenly distributed without the tendency of aggregation at C-terminal. It suggested that other mechanisms may be existed to contribute to hearing loss. The Gly-X-Y amino acid combination is considered to be one of the most significant peptide chain domains in the occurrence of *COL2A1*-related diseases. A glycine (Gly) appears for every three amino acid residues on the α1 chain. The structure composed of 330 Gly-X-Y repeats has the ability to bind receptors and other functional proteins, which is essential for the structural stability of type II collagen. Variations on the Gly-X-Y repeats would damage the triple-helical conformation and impair the intracellular transport of collagen, thus leading to over-modification of collagen and destruction of long bone development [14]. Corresponding to the enrichment of Gly-X-Y repeat in the COL2A1 protein, glycine substitutions constitute the predominant part of the *COL2A1* gene variation spectrum (305 of 405 variations till 2016) [1]. Before this study, all the six variations causing SEDC accompanied with SNHL were glycine substitutions. Glycine to serine substitutions, unlike glycine to nonserine residue substitutions, caused variable phenotypes [4, 23]. Some glycine to serine substitutions produced severe skeletal phenotypes, while others exclusively created mild skeletal phenotypes. In this study, the disease-causing variation was glycine to serine substitution and different individuals differ in the severity of spine and hip joint diseases, which was consistent with the feature of glycine-serine substitution.

## Conclusion

In conclusion, the hot mutation p.G504S in *COL2A1* gene was identified as the pathogenesis in this Chinese Li nationality family. It was the first report to find the correlation between p.G504S and atypical clinical phenotypes including sensorineural hearing loss, auditory ossicles deformity, retinal detachment, sacrum cracked and elbow and wrist joints deformity. Our findings would extend the phenotypic spectrum of SEDC and deepen clinicians' understanding of genotype–phenotype correlation of the disease.

## Supplementary Information

**Additional File 1**. **Supplementary tables. Supplementary Table 1.** The whole-exome sequencing parameters of the two patients. **Supplementary Table 2.** Filtration process for heterozygous variants. **Supplementary Table 3.** Variant pathogenicity analysis.

**Additional File 2.** Filtering flow of variants of patient II3.

**Additional File 3**. Filtering flow of variants of patient I2.

## Data Availability

The sequencing data used and/or analyzed during the current study are available at the following URL: https://www.ncbi.nlm.nih.gov/bioproject/739493 or in the BioProject database under accession number PRJNA739493.

## Abbreviations

SEDC: Spondyloepiphyseal dysplasia congenital
SNHL: Sensorineural hearing loss
WES: Whole exome sequencing
PTA: Pure-tone audiometry
Gly: Glycine
