# Supplementary material for: Discovery of sensorineural hearing loss and ossicle deformity in a Chinese Li nationality family with spondyloepiphyseal dysplasia congenita caused by p.G504S mutation of COL2A1
Source: BMC Med Genomics. 2021 Jun 28;14:170. doi: 10.1186/s12920-021-01020-y (PMC8240210; doi:10.1186/s12920-021-01020-y)
Supplement: Supplementary file 1 — Additional File 1. Supplementary tables. Supplementary Table 1. The whole-exome sequencing parameters of the two patients. Supplementary Table 2. Filtration process for heterozygous variants. Supplementary Table 3. Variant pathogenicity analysis. [file 12920_2021_1020_MOESM1_ESM.docx]

| Supplementary Table 1. The whole-exome sequencing parameters of the two patients | | | | | |
| --- | --- | --- | --- | --- | --- |
| **proband** | **length of target region (bp)** | **Target region map bases (Mbp)** | **Coverage (%)** | **Coverage at least 20× (%)** | **Mean Depth** |
| Ⅱ3 | 58682415 | 24428.01 | 99.73 | 98.72 | 138.90 |
| Ⅰ2 | 35735556 | 13226.12 | 99.52 | 98.89 | 180.52 |

| Supplementary Table 2. Filtration process for heterozygous variants | | |  |
| --- | --- | --- | --- |
| **Filter process** | **NO. of variants** | | |
|  | **Ⅱ3** | **Ⅰ2** | |
| Genotype frequency in 1000 human genome dataset, ExAC, ESP6500, GnomAD, BGI in-house Database≤0.01 | 336 | 384 | |
| Functional variations, including tran ablation/stop gained/stop lost/frameshift /missense/inframe insertion/deletion | 168 | 200 | |
| Predicted to be pathogenic by ClinVar Significance, SIFT, MutationTaster, Polyphen2, Phylop, GERP et al. | 52 | 62 | |
| Variants associated to phenotype/disease by checking for diseases databases | 1 | 1 | |

Supplementary Table 3. Variant pathogenicity analysis

| **Gene** | **NM_** | **AA** | **Zygosity** | **mutationtaster** | **SIFT** | **PROVEAN** | **ClinVar** | **Ens Condel Pred** | **MAF** | **PhyP/PhastCons** |
| --- | --- | --- | --- | --- | --- | --- | --- | --- | --- | --- |
| *COL2A1* | 001844.4 | p.G504S | Het | disease causing | Damaging | Deleterious | Pathogenic | deleterious | ≤0.01 | Conserved |
