# Supplementary figures and images for: Discovery of sensorineural hearing loss and ossicle deformity in a Chinese Li nationality family with spondyloepiphyseal dysplasia congenita caused by p.G504S mutation of COL2A1
Source: BMC Med Genomics. 2021 Jun 28;14:170. doi: 10.1186/s12920-021-01020-y (PMC8240210; doi:10.1186/s12920-021-01020-y)

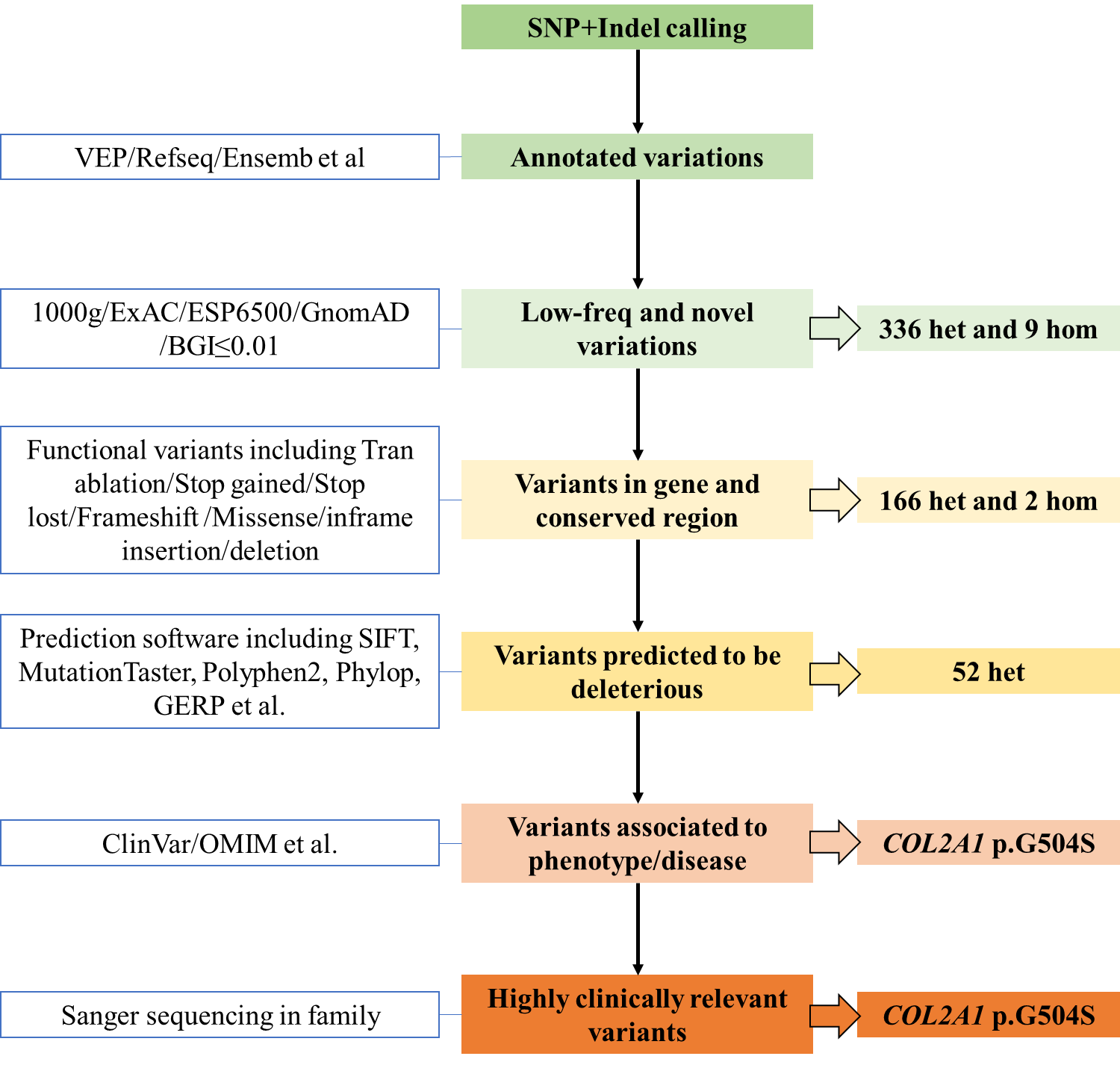

Supplement: Supplementary file 2 — Additional File 2. Filtering flow of variants of patient II3. [file 12920_2021_1020_MOESM2_ESM.png]

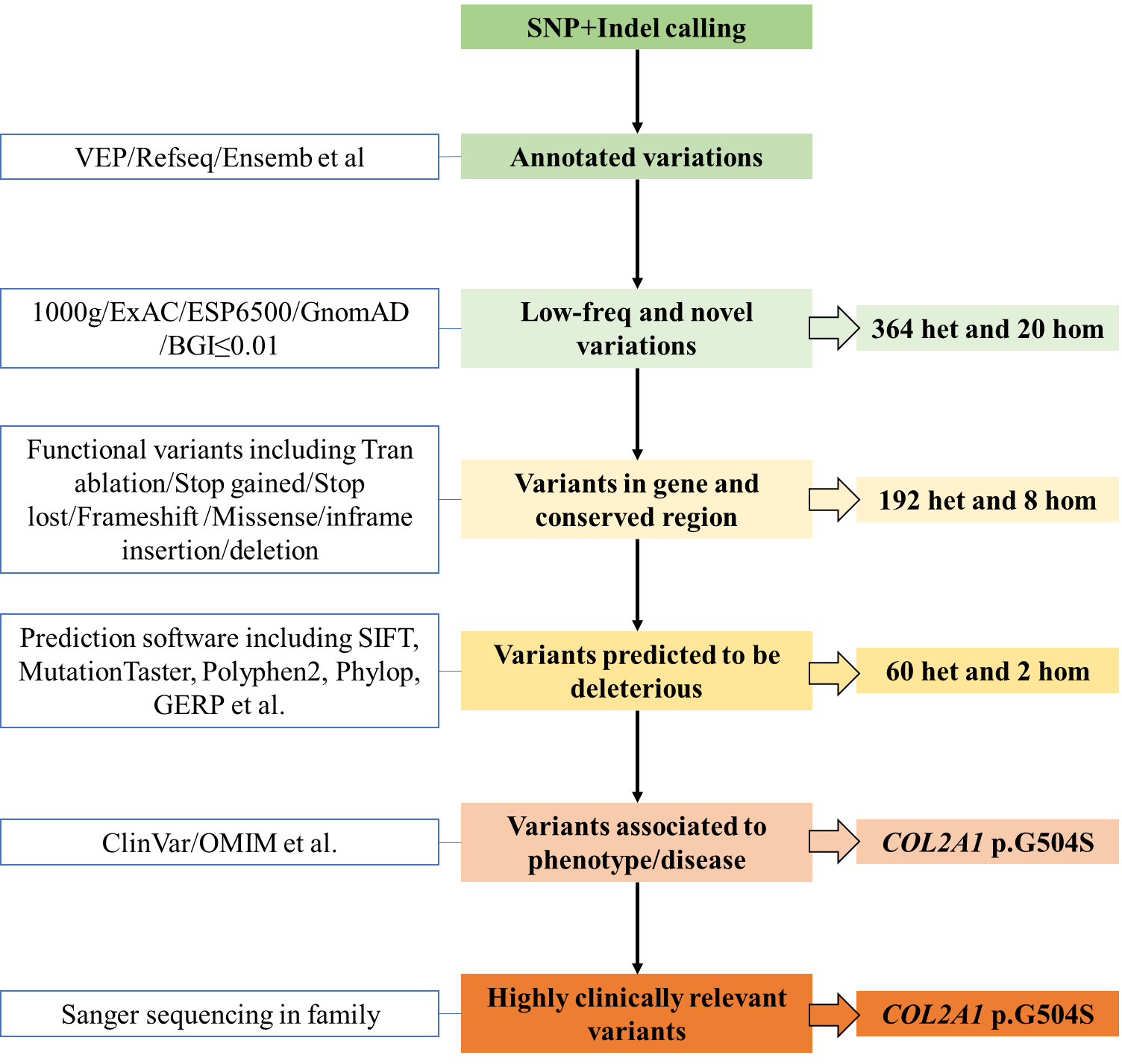

Supplement: Supplementary file 3 — Additional File 3. Filtering flow of variants of patient I2. [file 12920_2021_1020_MOESM3_ESM.png]
